# Metagenome-Wide Association Study and Machine Learning Prediction of Bulk Soil Microbiome and Crop Productivity

**DOI:** 10.3389/fmicb.2017.00519

**Published:** 2017-04-03

**Authors:** Hao-Xun Chang, James S. Haudenshield, Charles R. Bowen, Glen L. Hartman

**Affiliations:** ^1^Department of Crop Sciences, University of IllinoisUrbana, IL, USA; ^2^USDA—Agricultural Research ServiceUrbana, IL, USA

**Keywords:** machine learning, metagenome-wide association study, microbiome, nitrogen fixation, productivity, random forest, rhizobium, soybeans

## Abstract

Areas within an agricultural field in the same season often differ in crop productivity despite having the same cropping history, crop genotype, and management practices. One hypothesis is that abiotic or biotic factors in the soils differ between areas resulting in these productivity differences. In this study, bulk soil samples collected from a high and a low productivity area from within six agronomic fields in Illinois were quantified for abiotic and biotic characteristics. Extracted DNA from these bulk soil samples were shotgun sequenced. While logistic regression analyses resulted in no significant association between crop productivity and the 26 soil characteristics, principal coordinate analysis and constrained correspondence analysis showed crop productivity explained a major proportion of the taxa variance in the bulk soil microbiome. Metagenome-wide association studies (MWAS) identified more *Bradyrhizodium* and *Gammaproteobacteria* in higher productivity areas and more *Actinobacteria, Ascomycota, Planctomycetales*, and *Streptophyta* in lower productivity areas. Machine learning using a random forest method successfully predicted productivity based on the microbiome composition with the best accuracy of 0.79 at the order level. Our study showed that crop productivity differences were associated with bulk soil microbiome composition and highlighted several nitrogen utility-related taxa. We demonstrated the merit of MWAS and machine learning for the first time in a plant-microbiome study.

## Introduction

The soil microbiome has been a great interest for its potentials in improving plant nutrient utilization and suppressing soil-borne diseases (Müller et al., 2016). While abiotic soil characteristics such as pH, soil types, and trace elements can strongly influence a microbiome composition (Xu et al., 2009; Tkacz and Poole, 2015), biological factors such as plant species or genotypes can also influence a soil microbiome composition, resulting in taxonomic difference between genotypes (Peiffer et al., 2013; Lakshmanan, 2015). Accordingly, a soil microbiome composition could depend on abiotic and biotic factors, and variations in these factors may cause differences in crop productivity (Tkacz and Poole, 2015). Soybean [*Glycine max* (L.) Merr.] is one of the predominant crops grown in rotation with maize in agronomic fields of Illinois in the USA. Crop productivity differences in areas within a field have been noted by a number of producers, although the field itself may have the same cropping history, the same soybean genotype (cultivar), and the same management practices in a given season. A hypothesis for the crop productivity difference is that some beneficial and/or detrimental abiotic or biotic factors are unequally distributed in the bulk soils among areas in a field. A couple of studies have suggested the link between yield performances and soil microbiome differences for grape and millet (Debenport et al., 2015; Xu et al., 2015). This could also be the case for field crops.

In order to test this hypothesis, quantifications of a variety of abiotic soil characteristics and the taxa in a soil microbiome are needed. Abiotic soil characteristics can be measured by different chemical and physical analyses, but quantification of taxa can be technically challenging because of the complexity of the soil microbiome. Recent advances in metagenomics, which uses the power of next generation sequencing technology, provides for an approach to quantify taxa in the soil microbiome (Simon and Daniel, 2011). Metagenomics allows a direct detection and quantification of DNA sequences and bypasses the necessity to isolate the organisms, which might be rare in proportion and might be fastidious or unable to culture. Moreover, shotgun metagenomics avoids the concern of PCR amplification bias and provides functional annotation through gene enrichment analysis and pathway analysis (Sharpton, 2014). Although there are several technical challenges, such as sampling consistency from environments, DNA integrity and contamination, and bioinformatic difficulties in taxa annotation and quantification, the power of shotgun metagenomics has been demonstrated in several medical studies on finding associations between taxa in a microbiome and human diseases (Le Chatelier et al., 2013; Lakshmanan, 2015; Zhang et al., 2015). One approach to identify the association is using metagenome-wide association study (MWAS), which takes advantages of huge taxa data discovered using metagenomics and applies the concept of genome-wide association study (GWAS) for the association analysis. Instead of using single nucleotide polymorphisms (SNPs) as the explanatory variables, MWAS employs the abundance of a taxa (a metagenomic species or a metagenomic gene cluster) as the explanatory variables (Wang and Jia, 2016), and MWAS has been successfully used for several human diseases such as type 2 diabetes (Karlsson et al., 2013). Another advantage of the huge taxa data from a metagenomics is to use machine learning methods such as the Random Forest (RF) model or Support Vector Machine model, to integrate the abundance of metagenomic species for phenotypic prediction (Soueidan and Nikolski, 2016; Wang and Jia, 2016). Successful integrative studies for human microbiome and its association with human diseases have been demonstrated (Soueidan and Nikolski, 2016; Wang and Jia, 2016), but to our knowledge, the robustness of MWAS and machine learning has not yet been tested or applied on plant or soil metagenomic data.

Our goal in this study was to determine if abiotic or biotic factors associate with high and low crop productivity areas within agronomic fields. The objectives included the association between crop productivity with abiotic soil characteristics, and crop productivity with the abundance of metagenomic species based on the reference database from shotgun metagenomic analysis. We applied MWAS to find significant associations between taxa and productivity, and adapted machine learning using RF to predict productivity based on soil microbiome composition.

## Materials and methods

### Soil sampling and characterization

Soil samples were collected from six agronomic fields in Illinois (Figure S1). Ten soil core (2.5 cm diameter by 13 cm deep) subsamples were collected from each of two areas in each of the six fields. One area in the field was identified to be high in productivity and another area was identified to be low productivity based on the farmer production records. Samples for each bulk were taken from within an area of less than a 100-meter diameter circle. Samples were blended and divided. One part of each sample was frozen at −80°C and later lyophilized. The other part of each sample was used for CHN analysis (Microanalysis Laboratory, University of Illinois, Urbana, IL, U.S.A.), and were quantified for other 26 characteristics, including one biotic feature: the soybean cyst nematode (SCN) eggs counts, and 25 abiotic characteristics: latitude and longitude of sampling areas, percentage of clay, sand, and silt, 12 elements (B, Ca, Cu, Fe, Mg, Mn, N, P, K, Na, S, and Zn), percent saturation (PS) of five elements (PS.Ca, PS.H, PS.K, PS.Mg, and PS.Na), cation-exchange capacity (CEC), organic matter, and water pH (SGS North America Inc. Rutherford, NJ, U.S.A.). Pairwise Pearson's correlation was performed using R package “psych” version 1.6.6 (Revelle, 2016) in the R environment version 3.3.1 (R Core Team, 2015). The correlation plot was generated using R package “corrplot” version 0.77 with hierarchical clustering Ward.D2 method (Wei and Simko, 2016). Logistic regression was applied to understand the association between the crop productivity and the other 26 soil characteristics. Significance of Pearson's correlation and logistic regression were determined at *p*-value of 0.05.

### Shotgun sequencing and data archive

DNA was extracted from 200 mg subsamples of lyophilized, milled (model M20, Ika Works, Wilmington-NC) soil using the FastDNA SPIN Kit for Soil (MP Biomedicals. Solon, OH, U.S.A.) and further purified using the MicroElute DNA Clean-up Kit (Omega Bio-tek. Norcross, GA, U.S.A.). Twelve bulk soil DNA samples were deep shotgun sequenced in pairs through six lanes using Illumina HiSeq2000 (Roy J. Carver Biotechnology Center at the University of Illinois) using TruSeq SDS sequencing kit version 3 according to the manufacturers' protocols. The 12 shotgun sequencing data were deposited in MG-RAST server (Table 1).

**Table 1:** **Sampling information and metagenomic statistics of 12 bulk soil samples in Illinois**.

**Sample ID**	**MG-RAST ID**	**City (IL, USA)**	**Latitude**	**Longitude**	Productivity^*^	**Raw Reads**	**Filtered (%)**	**Post QC Reads**	**Alpha-diversity (species)**
1	4502923.3	Coulterville	38.11880	−89.37656	High	62,087,940	10.40	55,616,919	934.208
2	4502929.3	Coulterville	38.11917	−89.37632	Low	90,120,459	15.30	76,341,732	935.288
3	4502930.3	Greenfield	39.22606	−90.19410	High	124,404,467	8.60	113,747,535	908.248
4	4502931.3	Greenfield	39.22540	−90.19423	Low	93,849,320	4.90	89,256,102	921.719
5	4502932.3	Greenfield	39.22709	−90.19435	High	106,333,248	4.60	101,477,796	889.839
6	4502933.3	Greenfield	39.22728	−90.19357	Low	67,442,497	17.80	55,471,025	946.078
7	4502541.3	Auburn	39.35987	−89.45862	High	78,573,006	17.40	64,893,620	861.629
8	4502539.3	Auburn	39.35907	−89.45862	Low	67,333,977	7.70	62,155,903	897.977
9	4502926.3	Mansfield	40.13227	−88.27992	High	97,576,070	3.70	93,936,766	875.722
10	4502925.3	Mansfield	40.13185	−88.28042	Low	89,341,482	4.80	85,056,308	892.166
11	4502927.3	Urbana	40.04660	−88.13072	High	111,108,508	4.40	106,242,805	919.205
12	4502928.3	Urbana	40.04661	−88.12943	Low	81,221,533	4.40	77,618,380	912.711

* *Distance calculation between paired samling locations was 46 m (1 vs. 2), 74 m (3 vs. 4), 70 m (5 vs. 6), 89 m (7 vs. 8), 63 m (9 vs. 10), and 11 m (11 vs. 12) based on http://andrew.hedges.name/experiments/haversine/ and http://www.movable-type.co.uk/scripts/latlong.html. Samples were collected: 1–6 = 29 Oct. 2010, 7–8 = 01 Nov. 2010, and 9–12 = 02 Nov. 2010*.

### Metagenome analyses and metagenome-wide association study (MWAS)

Raw reads were uploaded to the MG-RAST server (Meyer et al., 2008), and quality-controlled reads were analyzed for taxa abundance using the best hit classification to the M5NR database (Wilke et al., 2012) and functional gene abundance using hierarchical classification to the Subsystems. Compared to the default parameters of MG-RAST server, higher stringent parameters were set at a minimum length of 30 nucleotides, a cutoff at 80% of identity, and a cutoff at an *E*-value of 1 × 10^−9^ in this study (Wilke et al., 2016). The first two principal coordinates (PCo1 and PCo2) generated by MG-RAST were also analyzed using Pearson's correlation to the 26 soil characteristics and the logistic regression to the crop productivity. Significance of Pearson's correlation and logistic regression were determined at *p*-value of 0.05. The highest-correlated soil characteristics to PCo1 and PCo2 (water pH and productivity) were labeled in the principal coordinate analysis (PCoA) plot generated in MG-RAST using normalized abundance. R package “mvtnorm” version 1.0–5 (Genz et al., 2016) and “ellipse” version 0.3–8 (Murdoch and Chow, 2013) were used to generate 90% confidence intervals for the high and low productivity samples. Constrained correspondence analysis or the canonical correspondence (CCA) with environmental vector fitting was performed using R package “vegan” version 2.4-1 (Oksanen et al., 2016). Since there is a multicollinearity problem among the 26 soil characteristics, the variance inflation factor (VIF) for each variable was estimated using R package “faraway” version 1.0.7 (Faraway, 2016). Because 12 soil samples cannot provide enough degree of freedom for a full model with 26 variables, variables (including water pH, crop productivity, and others with a VIF below 5) were used in vector fitting in the CCA, regarding as the VIF-based model. Akaike's information criterion (AIC) was applied to select useful soil characteristics in the AIC-based model for vector fitting. Permutation tests by marginal effects with 1,000 permutations were applied to estimate the significance of the AIC-based model and the VIF-based model. MWAS was performed to find significant associations between taxa and crop productivity using Wilcoxon rank sum test after filtering taxa with raw abundance below 12 counts across 12 soil samples from MG-RAST (Karlsson et al., 2013; Wang and Jia, 2016). Significant associations were determined at Benjamini-Hochberg adjusted *p*-value or false discovery rate (FDR) at α = 0.05.

### Machine learning using random forest (RF)

The RF machine learning was performed in R using “ranger” package (Wright and Ziegler, 2015). A total of 66 possible combinations, which included 10 samples as the training set and the remaining two samples as the testing set ($C_{2}^{12}$), were iterated in each run. Within each run, the number of trees (num.tree) was set at 500 to build the RF model. The number of variables/taxa that could be selected in each splitting node (mtry) was set in a range one tenth of the maximum taxon number at each taxonomic level (32 for phylum, 66 for class, 134 for order, 175 for family, 149 for genus, and 215 for species), and all of the 10 mtry parameters were run. The importance of each taxon was estimated using the Gini index, and the prediction accuracy was determined by $\frac{True\;Positive\;\left(TP\right)\;+\;True\;Negative\;\left(TN\right)}{TP+TN+False\;Positive+False\;Negative}$. A total of 100 runs for each mtry parameter were performed for each taxonomy hierarchy.

## Results

### Soil characteristics were not associated with crop productivity

**Figure 1:**
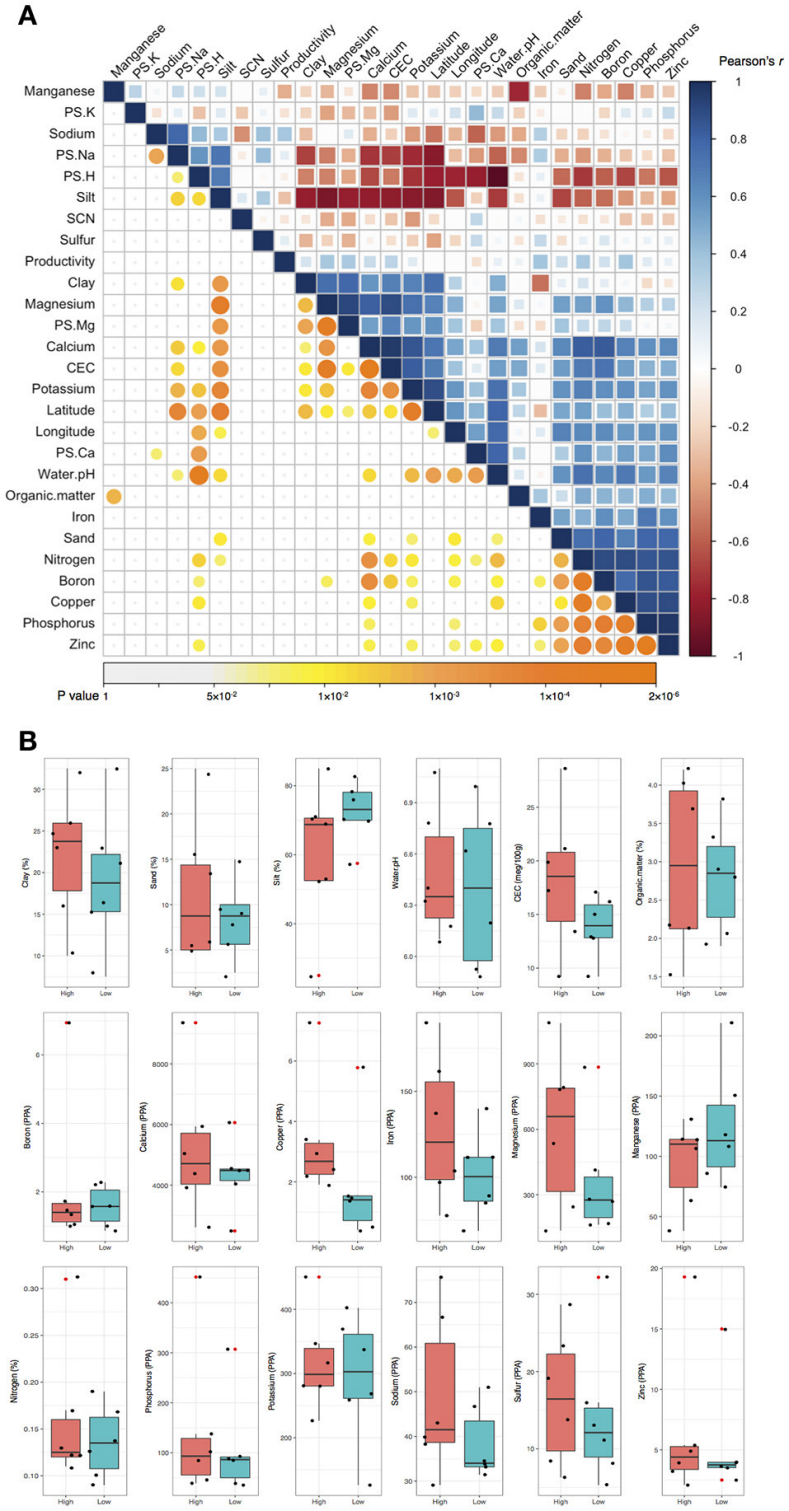
**Pairwise Pearson's correlation and logistic regression analyses. (A)** Pairwise Pearson's correlation analysis. The upper triangle displayed the Pearson's correlation coefficient (*r*) between each of the two soil characteristics. Blue and red color indicates positive and negative correlation, respectively. The color density and the square size reflect the scale of correlation. The lower triangle displayed the *p* value for each corresponding correlation. The color density and circle size demonstrate the significant level, and *p* values above 0.05 were regarded as insignificant and labeled in white color. None of the 26 soil characteristics was significantly correlated to crop productivity. **(B)** Logistic regression analysis. Crop productivity was assigned a response to each soil characteristics in the logistic regression. Black dots represent the 12 data of soil samples. CEC, cation-exchange capacity; PPA, pounds per acre; PS, percentage of saturation; SCN, soybean cyst nematode.

In the pairwise Pearson's correlation analysis, the 26 soil characteristics formed one negative correlated block and two positive correlated blocks (Figure 1A). In the negative correlated block, water pH significantly correlated with latitude, longitude, and several elements as well as clay and sand soil types. The first positive correlated block included several elements (such as boron, phosphorus, and zinc) and sand soil type. The second positive correlated block contained other elements (such as Ca, Mg, and K) with CEC, latitude, and longitude. A weaker positive correlation between the first and the second positive correlation block was significant as well. The preliminary analysis using Person's correlation demonstrated there was no significant association between crop productivity to any of the 26 soil characteristics (Figure 1A). To further confirm the observation, logistic regression was applied to understand the association between the binary crop productivity and each soil characteristic. The results of logistic regression support the observation of pairwise Pearson's correlation that none of the 26 soil characteristics was significantly associated with crop productivity (Figure 1B). The results together indicate that neither the abundance of SCN, which is the primary soil pathogen for soybean production in Illinois (Niblack and Riggs, 2015), nor the 25 soil characteristics was related to crop productivity difference. Other abiotic and biotic factors might associate with the productivity difference including the composition of bulk soil microbiome.

### Microbiome composition significantly associated with crop productivity

**Figure 2:**
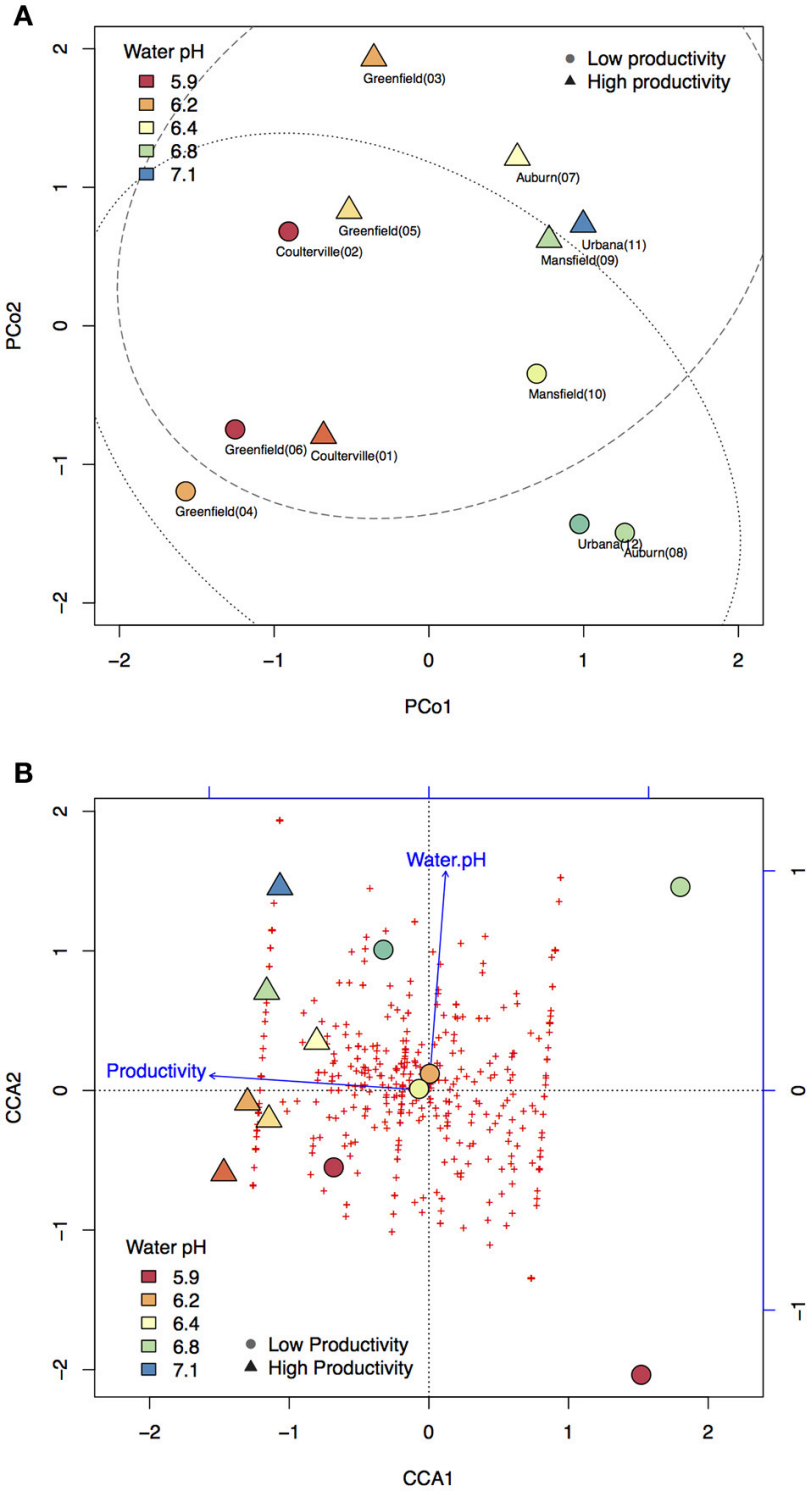
**Principal coordinate analysis (PCoA) and constrained correspondence analysis (CCA)**. Color panel indicates the water pH. Circle markers indicate samples with low productivity. Triangle markers indicate samples with high productivity. **(A)** Taxa variance was mostly explained by the principal coordinate 1 (PCo1) and PCo2. PCo1 has strong and significant correlation to water pH, and PCo2 has strong and significant correlation to crop productivity. Dotted line indicates the confidence interval of 90% for samples with low productivity. Dashed line indicates the confidence interval of 90% for samples with high productivity. **(B)** Taxa variance was mostly explained by the first and the second eigenvalue (CCA1 and CCA2). Each of the red crosses represents a taxon in the order level. Based on AIC-based model selection, crop productivity was the only significant variable required to explain the taxa variance. The addition of water pH as the second fitting vector resulted in a perpendicular direction to crop productivity, indicating the independency of these two variables.

To understand if microbiome compositions in bulk soil samples, shotgun sequencing was chosen to profile the soil microbiome. Pearson's correlation analysis indicated the highest variance (PCo1) in microbiome composition displayed a strong correlation to water pH (*p* = 0.0001). PCo1 separated acidic (*pH* < 6.3) and non-acidic (*pH* > 6.3) soil samples into two spaces, accounting for the largest 18% of taxa variance (Figure 2A). On the other hand, PCo2 explained second largest 13% of taxa variance. Moreover, PCo2 was the only factor that displayed a significant correlation to crop productivity (*p* = 0.0126) and the association was further confirmed by logistic regression (*p* = 0.0466). There were three samples (Greenfield 03, Auburn 07, and Urbana11) from high productivity areas and three samples (Greenfield 04, Auburn 08, Urbana 12) from low productivity areas located outside of the 90% confidence intervals (Figure 2A). Our observations in the PCoA were consistent to several publications that described the association between soil pH and microbiome (Rousk et al., 2010; Rascovan et al., 2016). However, the multicollinearity problem (such as the strong correlations between water pH and other characteristics) raises the possibility that water pH and crop productivity might be a confounding factor to PCo1 and PCo2, respectively.

In order to confirm the PCoA results, VIF-based model and AIC-based model were subjected to CCA with permutation supports. In the VIF-based model, six soil characteristics with VIF below 5 were included in the CCA model, including crop productivity (VIF: 1.05), organic matter (VIF: 1.19), PS.K (VIF: 1.26), SCN (VIF: 1.33), sulfur (VIF: 1.26), and water pH (VIF: 1.25) (Figure S2A). Among these six soil characteristics, permutation test identified crop productivity as the only significant explanatory variable (*p* = 0.014). When PCo1 and PCo2 were used as fitting vector in the VIF-based CCA, the result supported that PCo1 is closer to water pH while PCo2 is closer to crop productivity (Figure S2B). On the other hand, AIC-based model selection suggested the crop productivity as the only variable that needs to be included in the CCA to explain the taxa variance (AIC: 93.98, *p* = 0.055). The addition of water pH into the vector fitting CCA resulted in a perpendicular direction to crop productivity (Figure 2B), and when PCo1 and PCo2 were added into the CCA, a consistent result that PCo2 is closer to crop productivity can be observed (Figure S2C). ANOVA model comparison between VIF-based model (six explanatory variables) and AIC-based model (one explanatory variable) failed to reject the smaller AIC-based CCA model (*p* = 0.709). The results of CCA vector fitting indicated crop productivity is the major factor and explained 16% of taxa variance while water pH explained 8% of the taxa variance. Both PCoA and CCA demonstrated the taxa variance of microbiome composition was associated with crop productivity, which indicated the possibility that some taxa might vary between high and low productivity areas in the six fields.

### Metagenomic analyses and MWAS

**Figure 3:**
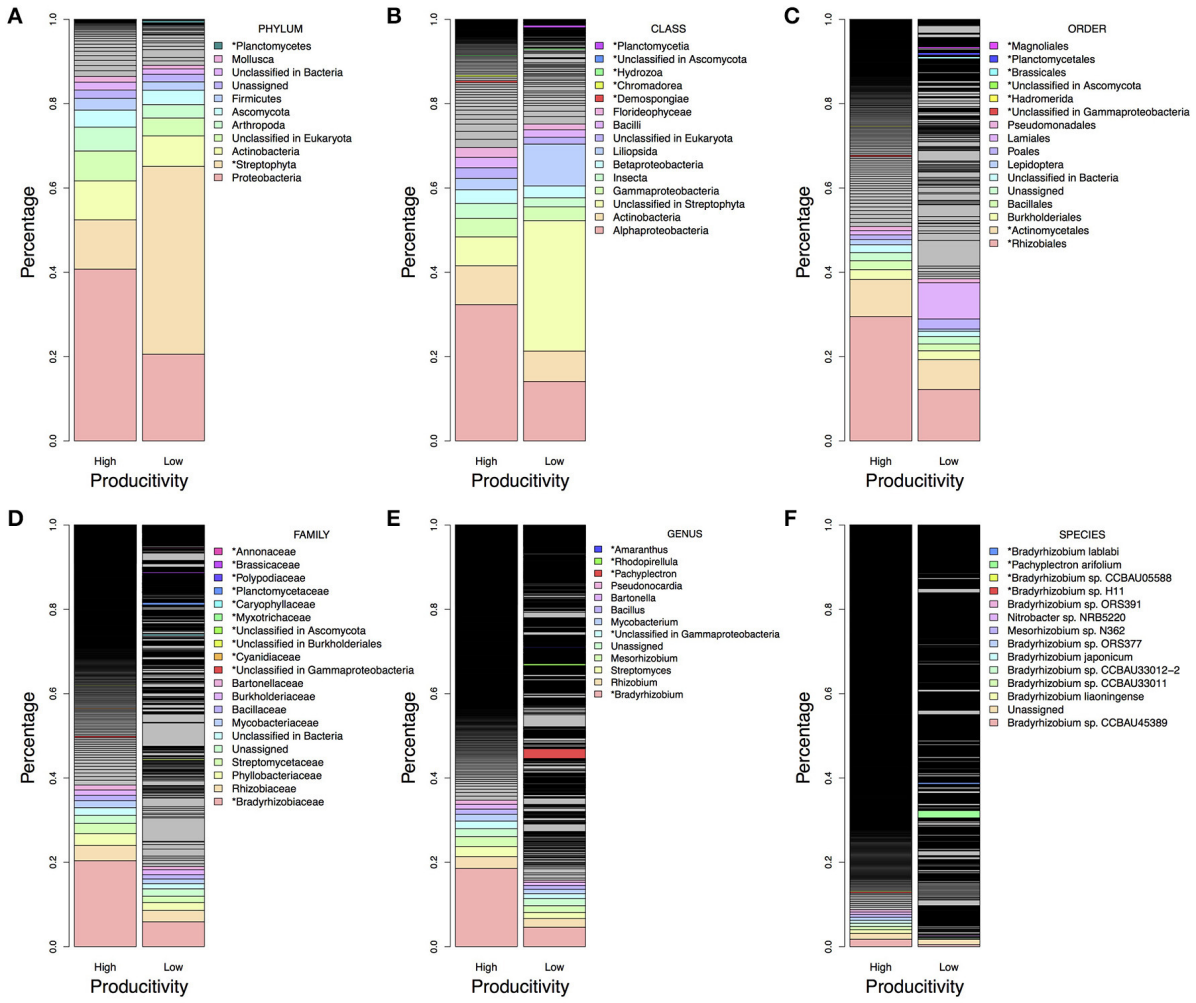
**Microbiome difference between high and low productivity areas**. The taxa proportion was the average of six soil samples from high and low productivity areas. The proportion was sorted from high to low abundance based on high productivity panel, and the top 10 abundant taxa were colored and labeled in light rainbow palette. Taxa with significant difference between high and low productivity areas were labeled with asterisks and with additional color palette. **(A)** Taxa in the phylum level. **(B)** Taxa in the class level. **(C)** Taxa in the order level. **(D)** Taxa in the family level. **(E)** Taxa in the genus level. **(F)** Taxa in the species level. Higher proportion of *Rhizobiales* order, *Bradyrhizobiaceae* family, *Bradyrhizobium* genus and some species presented in high productivity areas, while more *Steptophyta* and *Planctomycetes* could be found in low productivity areas.

There were more than 55 million sequencing reads for each sample that passed quality control and MG-RAST estimated an alpha-diversity around 861–935 for the 12 samples (Table 1). Welch independent two sample *t*-test suggested no significant difference for the alpha-diversity average from high and low productivity areas (*p* = 0.20). In order to identify what taxa in the bulk soil microbiome differ between the high and low productivity samples, we applied MWAS using Wilcoxon rank sum test. The abundance of a bacterial order *Planctomycetales* and a eukaryotic phylum *Streptophyta* were found significantly higher in low productivity areas. Both phyla can be detected more than three times at different hierarchies in the same taxonomy lineage (Table 2). Other significant taxa repeatedly identified by Wilcoxon rank sum test included a bacterial genus *Bradyrhizodium*, a bacterial class *Gammaproteobacteria*, an unclassified class in the fungal phylum *Ascomycota*, and an unclassified class in the eukaryotic phylum *Streptophyta* (Figure 3). The abundance of *Bradyrhizodium* and *Gammaproteobacteria* were generally higher in high productivity areas, while the abundance of *Ascomycota* was higher in low productivity areas (Figure 3, Table 2). In contrast to the success on MWAS, the association analysis between functional gene abundance and crop productivity failed to find anything significant results (data not shown).

**Table 2:** **Significant taxon abundance differed between high and low productivity areas using Wilcoxon rank sum test**.

**Domain**	**Phylum**	**Class**	**Order**	**Family**	**Genus**	**Species**	**High Prod^#^**	**Low Prod^#^**	FDR^*^
*Bacteria*	*Actinobacteria*	*Actinobacteria*	*Actinomycetales*				422	489	4.46E-02
*Bacteria*	***Planctomycetes***	***Planctomycetia***					1	38	2.51E-02
*Bacteria*	***Planctomycetes***	***Planctomycetia***	***Planctomycetales***				1	38	2.51E-02
*Bacteria*	***Planctomycetes***	***Planctomycetia***	***Planctomycetales***	*Planctomycetaceae*			1	38	2.51E-02
*Bacteria*	***Planctomycetes***	***Planctomycetia***	***Planctomycetales***	*Planctomycetaceae*	*Rhodopirellula*		0	31	9.47E-03
*Bacteria*	***Proteobacteria***	***Alphaproteobacteria***	***Rhizobiales***				1413	844	4.11E-02
*Bacteria*	***Proteobacteria***	***Alphaproteobacteria***	***Rhizobiales***	***Bradyrhizobiaceae***			983	410	2.60E-02
*Bacteria*	***Proteobacteria***	***Alphaproteobacteria***	***Rhizobiales***	***Bradyrhizobiaceae***	***Bradyrhizobium***		913	336	2.60E-02
*Bacteria*	***Proteobacteria***	***Alphaproteobacteria***	***Rhizobiales***	***Bradyrhizobiaceae***	***Bradyrhizobium***	*Bradyrhizobium lablabi*	1	40	2.08E-02
*Bacteria*	***Proteobacteria***	***Alphaproteobacteria***	***Rhizobiales***	***Bradyrhizobiaceae***	***Bradyrhizobium***	*Bradyrhizobium sp. CCBAU 05588*	19	2	3.61E-02
*Bacteria*	***Proteobacteria***	***Alphaproteobacteria***	***Rhizobiales***	***Bradyrhizobiaceae***	***Bradyrhizobium***	*Bradyrhizobium sp. H11*	21	4	3.61E-02
*Bacteria*	***Proteobacteria***	*Betaproteobacteria*	*Burkholderiales*	*Unclassified*			10	23	1.75E-02
*Bacteria*	***Proteobacteria***	***Gammaproteobacteria***	***Unclassified***				19	8	3.98E-02
*Bacteria*	***Proteobacteria***	***Gammaproteobacteria***	***Unclassified***	**Unclassified**			19	8	3.98E-02
*Bacteria*	***Proteobacteria***	***Gammaproteobacteria***	***Unclassified***	**Unclassified**	**Unclassified**		19	7	1.53E-02
*Eukaryota*	***Ascomycota***	*Leotiomycetes*	*Unclassified*	*Myxotrichaceae*			3	10	2.48E-02
*Eukaryota*	***Ascomycota***	***Unclassified***					3	9	2.39E-02
*Eukaryota*	***Ascomycota***	***Unclassified***	*Unclassified*				3	9	2.39E-02
*Eukaryota*	***Ascomycota***	***Unclassified***	*Unclassified*	*Unclassified*			3	9	2.39E-02
*Eukaryota*	*Cnidaria*	*Hydrozoa*					13	26	4.27E-02
*Eukaryota*	*Nematoda*	*Chromadorea*					21	8	3.47E-02
*Eukaryota*	*Porifera*	*Demospongiae*					23	6	1.87E-02
*Eukaryota*	*Porifera*	*Demospongiae*	*Hadromerida*				13	1	1.46E-02
*Eukaryota*	***Streptophyta***	*Liliopsida*	*Asparagales*	*Orchidaceae*	*Pachyplectron*		2	168	2.60E-02
*Eukaryota*	***Streptophyta***	*Liliopsida*	*Asparagales*	*Orchidaceae*	*Pachyplectron*	*Pachyplectron arifolium*	2	168	2.60E-02
*Eukaryota*	***Streptophyta***	*Polypodiopsida*	*Polypodiales*	*Polypodiaceae*			1	11	2.48E-02
*Eukaryota*	***Streptophyta***	***Unclassified***	*Brassicales*				1	33	2.05E-02
*Eukaryota*	***Streptophyta***	***Unclassified***	*Brassicales*	*Brassicaceae*			0	20	2.84E-02
*Eukaryota*	***Streptophyta***	***Unclassified***	*Caryophyllales*	*Amaranthaceae*	*Amaranthus*		0	13	2.69E-02
*Eukaryota*	***Streptophyta***	***Unclassified***	*Caryophyllales*	*Caryophyllaceae*			2	27	1.85E-02
*Eukaryota*	***Streptophyta***	***Unclassified***	*Magnoliales*				1	26	4.61E-03
*Eukaryota*	***Streptophyta***	***Unclassified***	*Magnoliales*	*Annonaceae*			0	14	2.80E-02
*Eukaryota*	*Unclassified*	*Bangiophyceae*	*Cyanidiales*	*Cyanidiaceae*			13	1	1.98E-02

* *FDR, False Discovery Rate*.

# *High Prod: the sum of a taxon abundance in the six soil samples from high productivity areas. Low Prod: the sum of a taxon abundance in the six soil samples from low productivity areas*.

*Bold, taxa detected to be significant for at least three times in MWAS*.

### Productivity prediction by using RF machine learning

**Figure 4:**
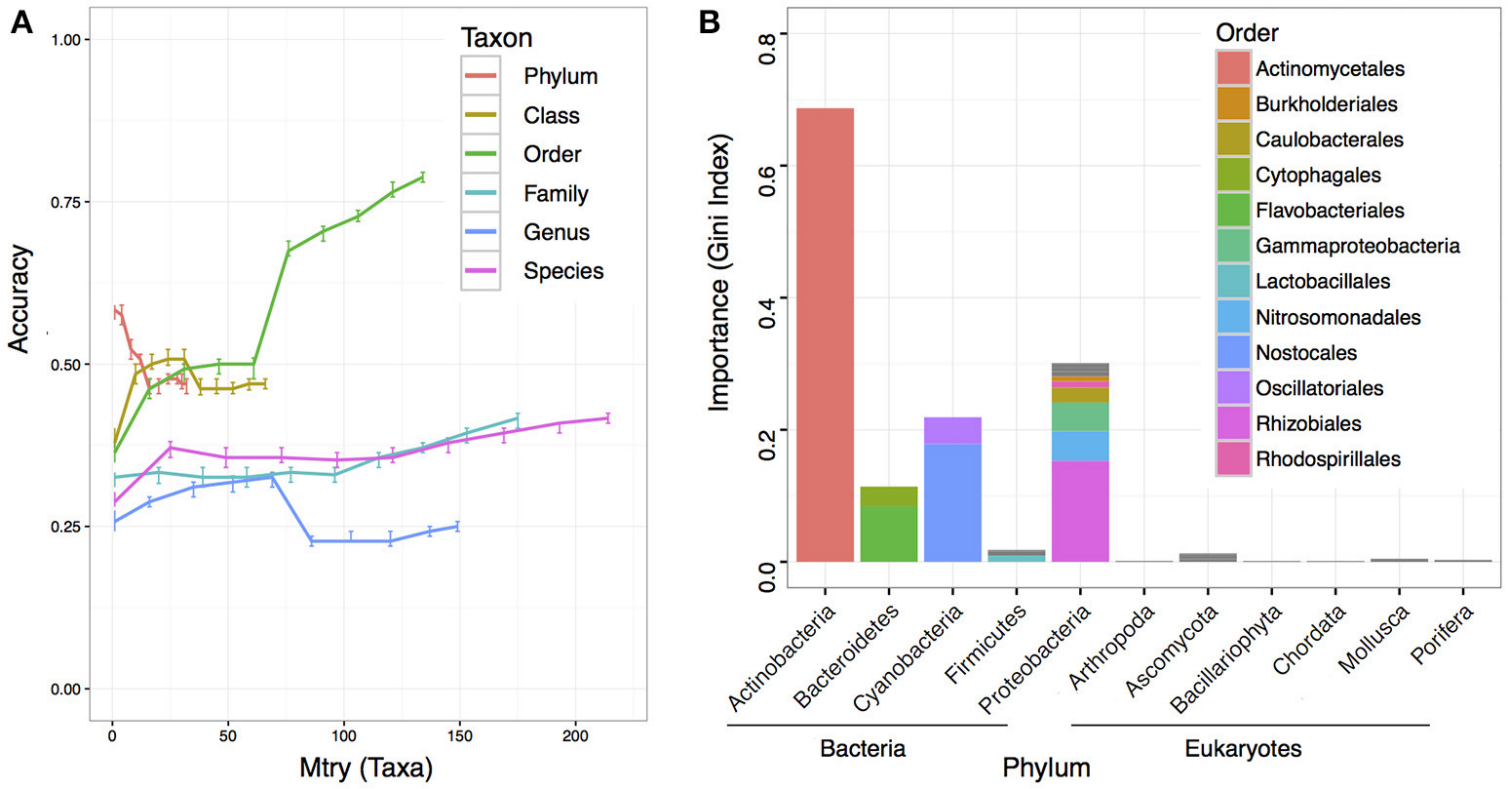
**Prediction accuracy of machine learning using random forest**. A total of 500 trees were computed in each run, and 100 runs were performed to reach an average for each point at each taxonomy hierarchy. **(A)** The y-axis indicates the accuracy value. Higher accuracy indicates better prediction. The x-axis indicates the number of variables/taxa allowed to be randomly selected in each split node. The bars at each point indicate the interquartile of the data point. **(B)** The importance of each taxon in the RF prediction at the order level. A total of 134 taxa were included in the model. The top 12 influential taxa were labeled in color and grouped by domain and phylum.

To understand if the microbiome composition in the bulk soils could be informative to predict crop productivity, we adapted RF machine learning and estimated the prediction accuracy at each taxonomic level with 10 different variables/taxa (mtry) included in the RF model. While most of the predictions had low accuracies, we found at the order level with all variables in the model reached the best prediction accuracy at 0.787 (Figure 4A). We further computed the taxon importance assigned by the RF model at the order level, and the results indicated most important taxon was the *Actinomycetiales*, and followed by *Nostocales* and *Rhizobiales* (Figure 4B). While the *Nostocales* in the *Cyanobacteria* phylum was only found to be important by RF machine learning, both *Actinomycetiales* and *Rhizobiales* were identified to be significant in the MWAS (Table 2). In addition to *Rhizobiales*, other taxa such as an unclassified order under the *Gammaproteobacteria* in the *Proteobacteria* phylum was also found to be important by MWAS and RF machine learning (Figure 4B).

## Discussion

Crop productivity is a quantitative trait determined by a variety of factors (Van Roekel et al., 2015). Abiotic soil characteristics such as water and nitrogen availability are well-known for being productivity-limiting factors (Durán et al., 2014), and weather conditions such as rainfall or temperature may significantly impact on crop productivity. On the other hand, biotic features such as pathogen, pest (Hartman et al., 2015), and beneficial symbiosis such as root nodulation (Tkacz and Poole, 2015) are also involved in yield performance. Moreover, an important biotic feature is the genetics of the crop variety (the genotype), which includes the genetics of the photosynthesis and productivity performance (Dhanapal et al., 2016; Li et al., 2016), the genetics of water and nitrogen utilizing efficiency (Dhanapal et al., 2015; Chen et al., 2016), the genetics of disease and pest resistance (Chang et al., 2016; Revelle, 2016), and the genetic influence on structuring the rhizosphere microbiome (Jin et al., 2009; Babujia et al., 2016; de Almeida Lopes et al., 2016). In our study, we discovered additional factors underlying crop productivity when the above factors were identical or similar. Our experimental design ensured each pair of two areas in the same agronomic field (with no known difference in environmental conditions such as rainfall) received the same management by the farmers (with identical crop genetic variety and agricultural applications such as fertilization) at each of the six locations in Illinois, and because there were known differences in diseases or pests reported in the sampling season between the two areas, we hypothesized that bulk soils with some unevenly distributed abiotic or biotic factors might be a cause of crop productivity difference.

Twenty-six soil characteristics were quantified in the soil samples; however, none of these displayed significant correlations to crop productivity. Nonetheless, other abiotic characteristics such as soil physical compaction, soil moisture, or drainage difference between areas should be considered as potential abiotic factors because the soil system is much more complicated than the 26 characteristics included in the studies. While the 26 soil characteristics led to no significant result, PCoA and CCA both suggested crop productivity was an important explanatory variable that accounted for the taxa variance of microbiome composition. In other word, microbial difference in the bulk soil samples might associate with crop productivity. To further understand which taxa in the microbiome associated with crop productivity difference, MWAS was applied to dissect the microbiome by individual taxa at different taxonomy levels (from Phylum to Species). Three bacterial taxa (*Bradyrhizodium, Gammaproteobacteria*, and *Planctomycetales*) and two eukaryotic taxa (*Ascomycota*, and *Streptophyta*) were found significant for at least three times in the same hierarchical lineage. Interestingly, most of these taxa were related to nitrogen utility one way or another. It was suggested that the abundance of *Proteobacteria* was higher when nitrogen is more available (Fierer et al., 2012), and indeed, we observed higher abundance of *Proteobacteria* in the higher productivity areas (Figure 4). Both *Bradyrhizodium* and *Gammaproteobacteria* belong to *Proteobacteria* phylum, and both taxa related to nodulation. Bacteria in the *Bradyrhizodium* genus are well known for their symbiosis roles with legumes to fix nitrogen and benefit crop productivity (Durán et al., 2014). The interactions between soybean and different *Bradyrhizodium* strains on crop productivity were shown to be significant (Zimmer et al., 2016). The distribution and diversity of *Bradyrhizodium* strains in the U.S.A. were also reported to vary geographically (Shiro et al., 2013). Most bacteria in the *Bradyrhizodium* genus were reported to have nitrogen-fixation genes (Durán et al., 2014), and their ability to fix nitrogen for more than half of soybean N demand was recognized (Salvagiotti et al., 2008). Although bacteria in the *Gammaproteobacteria* class may not have independent nitrogen fixation ability, some bacteria such as those in the genus *Klebsiella* were able to colonize peanut nodules in the presence of *Bradyrhizodium* species (Ibá-ez et al., 2009), and some were assumed to be disease-suppressive or health-promotive (Berendsen et al., 2012). The abundances of beneficial rhizobia (*Bradyrhizodium* and *Gammaproteobacteria*) were generally higher in the high productivity areas (Table 2). On the other hand, the order *Planctomycetales* belongs to a special group of bacteria that contain no peptidoglycan and mainly reproduce by budding. Classification for *Planctomycetes* situates the group in between Bacteria and Archea because some *Planctomycetes* have eukaryotic characteristics, such as a membrane-bound nucleoid and the ability to synthesize sterol. Moreover, some *Planctomycetes* performs anaerobic oxidation of ammonium to dinitrogen in specialized vesicles called anamoxosomes, which might reduce nitrogen availability in the bulk soils (Fuerst and Sagulenko, 2011). While *Streptophyta* is the phylum of land plants and algae that may directly compete for nitrogen availability with crops (Leliaert et al., 2012; Becker, 2013), *Ascomycota* is the largest fungal phylum that contains diverse soil-borne plant pathogenic fungi, but the phylum also contains many non-pathogens and additional studies that focus on what taxa are beneficial or detrimental for crop productivity are required as direct evidence. The *Actinomycetales* order also contains plant pathogens such as potato scab pathogen, *Streptomyces scabies*. While *Actinomycetales* was detected significantly only at the order level (not significant for *Actinobacteria* phylum and class), the group of bacteria was weighted as the most important taxon in RF machine learning. Although the abundance of *Actinomycetales* was higher in low productivity areas similar to the abundance of *Ascomycota, Planctomycetales*, and *Streptophyta* (Table 2), which gives an intuition that *Actinomycetales* may be detrimental to crop health, some studies reported co-inoculation benefits of *Actinomycetes* with *Bradyrhizodium japonicum* that promoted soybean growth (Soe et al., 2012; Nimnoi et al., 2014). As the *Actinomycetales* order still includes too many taxa to be conclusive, additional studies that focus on what taxa in the *Actinomycetales* order are beneficial or detrimental for crop productivity are required as direct evidence.

**Figure 5:**
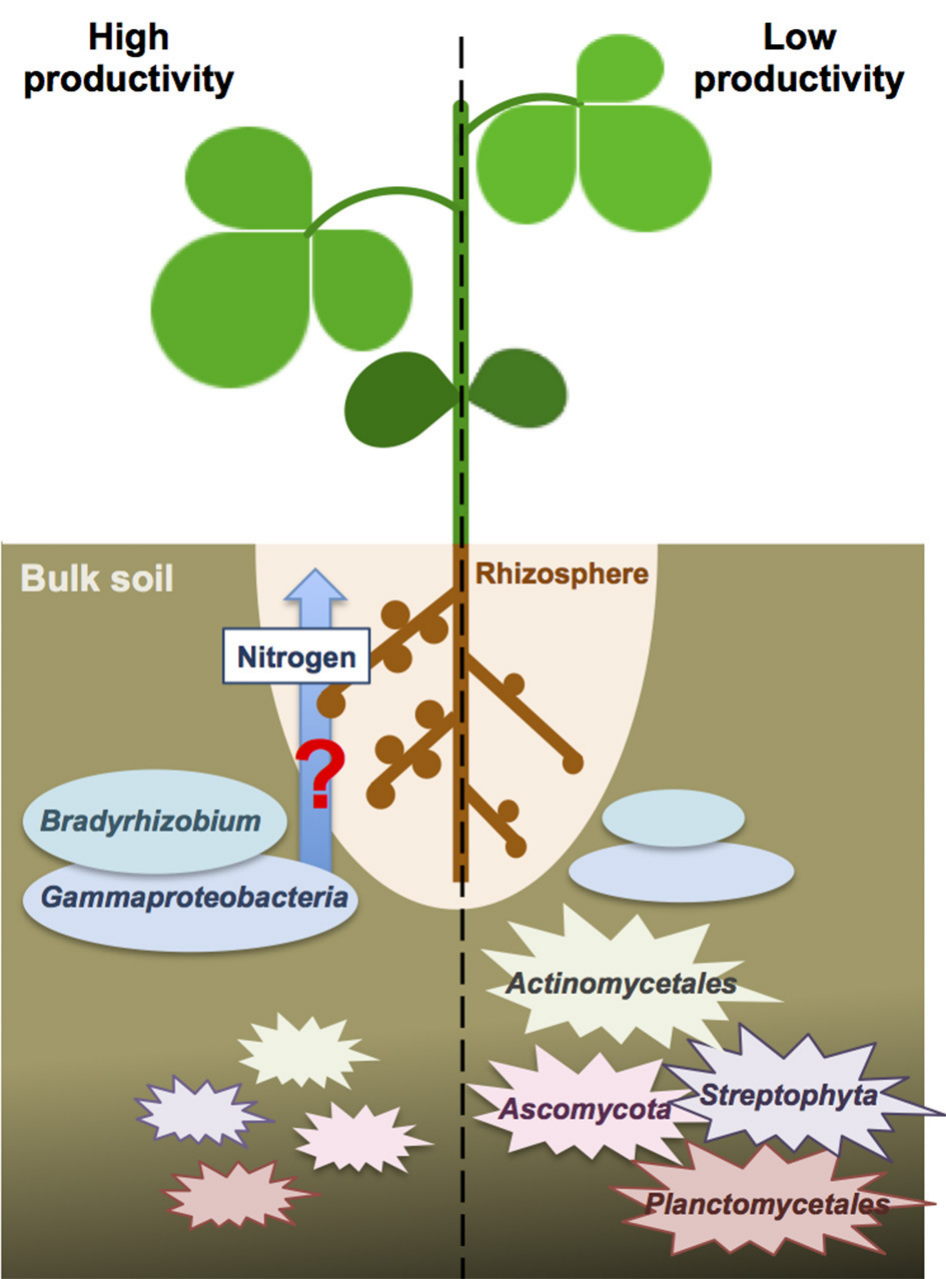
**The speculative scheme for the interactions between crop productivity and bulk soil microbiome**. The beneficial taxa of *Bradyrhizodium* and *Gammaproteobacteria* may interact with crops regarding nitrogen fixation and nodule formation to enhance nitrogen availability. On the other hand, higher abundance of *Steptophyta* and *Planctomycetes* in the bulk soil may compete nitrogen with crops and reduce nitrogen availability. Higher abundance of *Actinomycetales* and *Ascomycota* may increase biotic stress to crops.

Because the nitrogen content in the bulk soils was not significantly different between high and low productivity areas (Welch independent two sample *t*-test, *p* = 0.53), we speculated the different crop productivity might associate with nitrogen fixation activities inside the nodules. Higher abundance of *Bradyrhizodium* and *Gammaproteobacteria* may contribute to higher abundance of beneficial rhizobia in the rhizosphere. Indeed, it has been proposed that the rhizosphere microbiome of soybean was specialized from bulk soil microbiome to enhance soybean growth and nutrient utilities (Mendes et al., 2014). Unfortunately, the association results between functional gene abundance and crop productivity failed to identify any significant result, nor for nitrogen metabolism-related genes. But because DNA-based metagenomic study does not provide direct expression evidence, even if nitrogen metabolism-related genes appeared to be more abundant, a meta-transcriptomic study is still needed to ensure nitrogen metabolism-related genes indeed have differential expression in one condition over another. Advanced studies focus on finding evidence for recruitment of these beneficial rhizobia from the bulk soil into the rhizophere, and finding proofs for these benefitial rhizobia provide better nitrogen fixation or stimulate more nodules will provide a new insight to the crop productivity difference in a field (Figure 5).

In addition to identify taxa relating to crop productivity difference, our study applied machine learning prediction for the first time using soil microbiome composition. The result demonstrated that microbiome composition indeed could be useful for crop productivity prediction. While the prediction model with a small training set resulted in lower accuracy compare to machine learning prediction in human diseases (generally included 100–300 samples; Pasolli et al., 2016), we expect the accuracy would be improved with a larger sample size. Nonetheless, because soil microbiome could be far more complicated and diverse than human microbiome, limited sequencing depth to detect rare taxa and the reproducibility under the challenge of highly varied environmental factors will be the technical bottlenecks.

## Conclusion

We identified four groups of bacteria and two groups of eukaryotes that were significantly associated with crop productivity. The use of a RF model successfully predicted crop productivity at an accuracy of 0.79. With the active progress in metagenome annotation, statistical comparison, and computational power to handle high dimensional data, we expect that MWAS and machine learning will provide a new understanding on how microbial communities interact with crops and deliver direct benefits to agriculture.

## Disclosure

Trade and manufacturers' names are necessary to report factually on available data; however, the USDA neither guarantees nor warrants the standard of the product, and the use of the name by USDA implies no approval of the product to the exclusion of others that may also be suitable.

## Author contributions

HC: Analyzed and interpreted the data and developed the draft of the manuscript; JH: Interpreted the data and helped to write the manuscript; CB: Collected raw materials for experiment and helped to write the manuscript; GH: Coordinated the research and helped to write the manuscript.

## Funding

Research reported in this publication was supported by the Illinois Soybean Board and the USDA Agricultural Research Service.

### Conflict of interest statement

The authors declare that the research was conducted in the absence of any commercial or financial relationships that could be construed as a potential conflict of interest.

## Supplementary material

Figure S1 **Sampling location in the Illinois state**. A collaborator in each location (two collaborators in the Greensfield) provided two samples from each field, one with high productivity and one with low productivity.

Figure S2 **Constrained correspondence analysis (CCA). (A)** VIF-based CCA model, which includes six soil characteristics as explanatory variables. **(B)** VIF-based CCA model with PCo1 and PCo2 included. **(C)** AIC-based CCA model with PCo1 and PCo2 included.
