# Supplementary figures and images for: Metagenome-Wide Association Study and Machine Learning Prediction of Bulk Soil Microbiome and Crop Productivity
Source: Front Microbiol. 2017 Apr 3;8:519. doi: 10.3389/fmicb.2017.00519 (PMC5378059; doi:10.3389/fmicb.2017.00519)

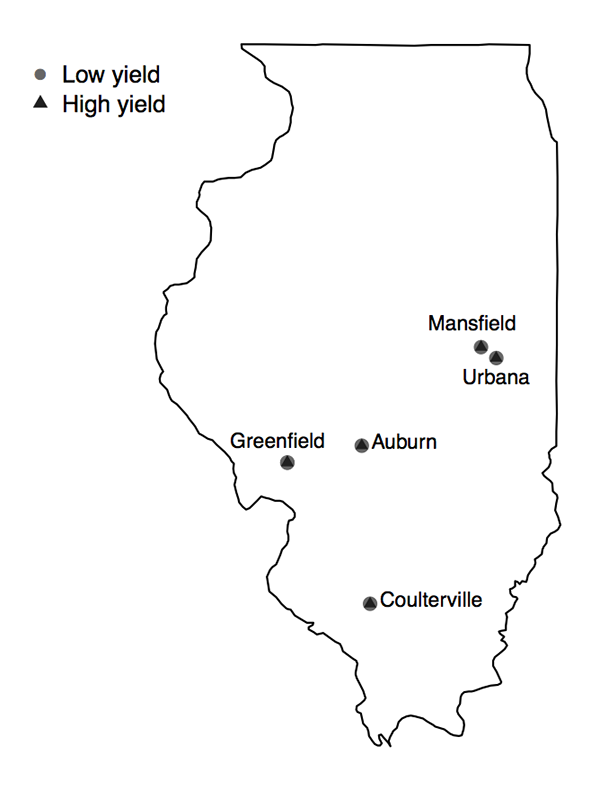

Supplement: Figure S1 — Sampling location in the Illinois state. A collaborator in each location (two collaborators in the Greensfield) provided two samples from each field, one with high productivity and one with low productivity. [file Image1.TIFF]

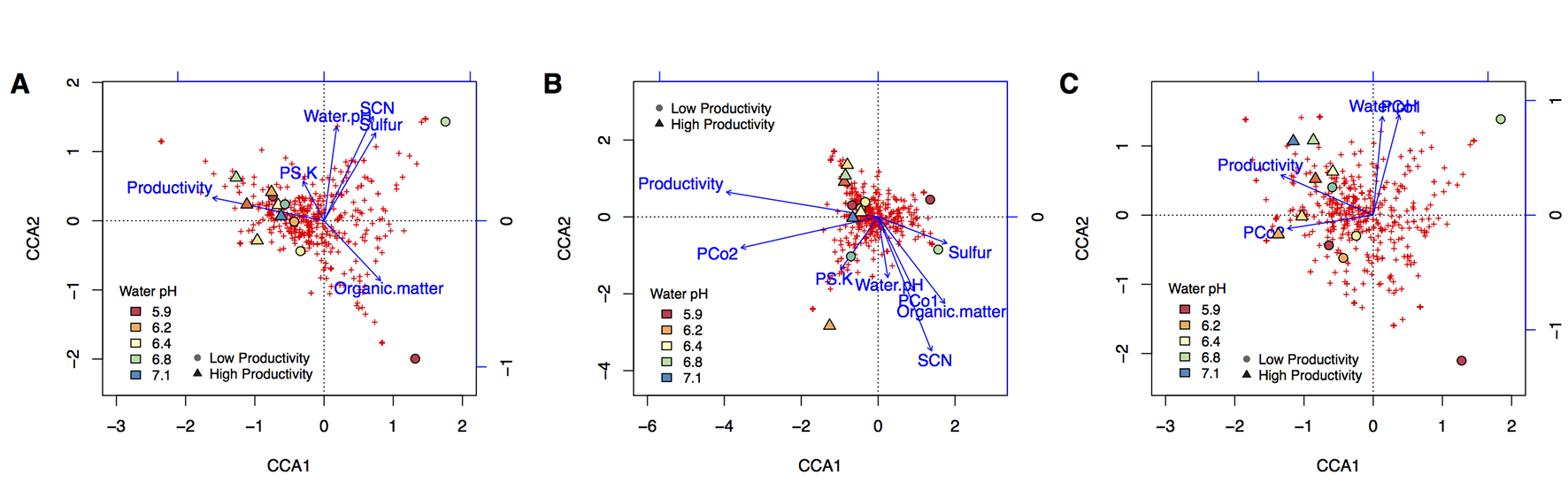

Supplement: Figure S2 — Constrained correspondence analysis (CCA). (A) VIF-based CCA model, which includes six soil characteristics as explanatory variables. (B) VIF-based CCA model with PCo1 and PCo2 included. (C) AIC-based CCA model with PCo1 and PCo2 included. [file Image2.TIFF]
